# An Interpretable Chest X-ray Classification Framework Using Prototype Memory and Counterfactual Consistency

**DOI:** 10.7759/cureus.103134

**Published:** 2026-02-06

**Authors:** Ling-Feng Chiang

**Affiliations:** 1 Internet of Things (IoT) Engineering and Applications, Yu Da University of Science and Technology, Miaoli County, TWN

**Keywords:** chest x-ray classification, counterfactual consistency, interpretable deep learning, medical image reliability, prototype memory network, pulmonary imaging

## Abstract

Chest X-ray (CXR) interpretation requires recognition of subtle and heterogenous radiographic patterns, yet conventional deep learning models often rely on spurious image cues that lack anatomical or pathological relevance. This study presents CXR-NeXus, an interpretable chest X-ray classification framework designed to promote explicit visual reasoning and reliable decision behavior under weak supervision. The proposed method integrates prototype memory with counterfactual consistency to constrain model predictions to clinically meaningful pulmonary evidence.

CXR-NeXus learns multiple class-specific prototypes that represent diverse radiographic phenotypes for COVID-19, pneumonia, tuberculosis, and normal cases. Each prediction is supported by similarity to representative prototype patterns, enabling transparent “this-looks-like-that” explanations at the image level. To further ensure causal dependence on pathological evidence, counterfactual chest X-rays are generated by Grad-CAM-guided lesion suppression constrained within coarse lung regions. The model is explicitly trained to reduce disease confidence when lesion evidence is removed, aligning prediction behavior with clinical intuition.

In addition, an evidence-alignment regularization is introduced to penalize extra-pulmonary saliency and to localize attention changes induced by counterfactual perturbations. The overall learning objective jointly optimizes classification performance, margin-based prototype separation, counterfactual consistency, and anatomically plausible attention, without requiring pixel-level lesion annotations. Experiments on a four-class chest X-ray dataset demonstrate that the proposed framework improves macro-average F1 score, Receiver Operating Characteristic - Area Under the Curve (ROC-AUC), specificity, and probability calibration compared with strong baselines, while substantially reducing reliance on spurious visual cues. These results suggest that combining prototype-level semantic anchoring with counterfactual-driven visual reasoning provides a practical and interpretable solution for reliable chest X-ray analysis.

## Introduction

Chest X-ray (CXR) is the most widely accessible imaging modality for respiratory diseases and remains fundamental to clinical triage and public health surveillance [1,2]. Among thoracic conditions, four diagnostic categories - COVID-19, pneumonia, tuberculosis (TB), and normal findings - span acute viral infection, heterogeneous inflammatory disease, chronic communicable infection, and physiological baseline. Together, these categories reflect clinically meaningful strata associated with distinct treatment urgency, infection-control measures, and resource allocation priorities [3,4]. COVID-19 and pneumonia may rapidly progress to hypoxemia and respiratory failure, whereas TB requires early detection to prevent community transmission and long-term morbidity [1,2]. Accurate identification of normal CXRs is equally critical, serving as a negative anchor to reduce overdiagnosis and unnecessary isolation or hospitalization [3,4].

Despite rapid advances in deep learning-based CXR analysis, automated diagnostic systems continue to face challenges in generalizability, interpretability, and clinical auditability [3,4]. Radiographic manifestations of COVID-19, pneumonia, and TB partially overlap and often lack disease-specific signatures, increasing the risk that high-performing classifiers rely on dataset bias or non-pulmonary cues rather than true pathological evidence [5,6]. COVID-19 commonly presents with diffuse ground-glass opacities, pneumonia may show lobar consolidation or interstitial changes, and TB often manifests upper-lobe fibrosis or cavitation; however, these patterns are neither exclusive nor consistently present [7,8]. Such ambiguity makes multi-class CXR classification particularly vulnerable to shortcut learning and undermines trust when models are deployed across institutions or imaging protocols [5,6].

Early studies demonstrated the feasibility of using convolutional neural networks (CNNs) to automate TB detection and pneumonia classification, particularly in high-burden or resource-limited settings [1,2]. Subsequent work expanded toward multi-disease CXR classification to better align with clinical reporting and triage workflows [3,4]. Large-scale public datasets - including ChestXray8, CheXpert, MIMIC-CXR, PadChest, VinDr-CXR, and COVID-19-specific repositories - have further accelerated benchmarking and methodological comparison [9-16]. However, most of these datasets rely on weak supervision extracted from radiology reports, introducing label noise, class imbalance, and domain bias [5,6]. Moreover, many comparative studies emphasize overall accuracy or area under the curve while providing limited analysis of calibration, robustness, or failure modes under distribution shift [5,6].

Interpretability has therefore become a central concern in CXR-based AI systems. Gradient-based saliency methods such as Grad-CAM offer post hoc visualization of model attention but do not establish a causal link between highlighted regions and prediction behavior [17]. Prototype-based approaches introduce “this-looks-like-that” reasoning by associating predictions with representative feature patterns, improving transparency but remaining sensitive to spurious cues and lacking mechanisms to verify evidence dependence [18,19]. Explanation-aligned training strategies attempt to constrain models to use predefined regions or masks deemed clinically relevant, yet they often require additional annotations or priors that are not readily available at scale [20].

More recently, counterfactual explanation methods have been proposed to probe decision boundaries by modifying input evidence and observing changes in model predictions [21-23]. These approaches highlight the importance of enforcing predictable model behavior when salient evidence is removed [21-23]. In parallel, self-supervised representation learning has shown promise in improving downstream CXR classification performance under limited or noisy labels, although interpretability and clinical auditability are typically not central objectives [24]. Large curated releases such as MIMIC-CXR-JPG further enable scalable research but introduce additional considerations regarding preprocessing effects and clinical fidelity [25]. Task-specific studies focusing on COVID-19 versus non-COVID pneumonia classification reflect realistic clinical decision scenarios, yet they often exclude TB, rely on narrow data sources, and provide limited evaluation of explanation quality or calibration [7,8].

Collectively, these lines of work reveal a persistent gap between high classification performance and clinically trustworthy behavior [5,6]. Existing models may achieve strong metrics while failing to demonstrate that their decisions are grounded in appropriate pulmonary evidence, calibrated across disease categories, and robust against confounding factors [5,6]. This limitation is especially pronounced in multi-class settings that combine acute, chronic, and normal conditions, where reliable discrimination requires both sensitivity to subtle lesions and restraint against false positives.

To address these challenges, we propose CXR-NeXus, a weakly supervised framework designed to integrate classification performance with causal interpretability and auditability. The core objective of CXR-NeXus is to explicitly constrain learning such that model decisions depend on relevant pulmonary evidence and exhibit predictable behavior when that evidence is removed. By combining margin-based classification, prototype memory, and counterfactual consistency under anatomical priors, the framework aims to improve not only accuracy but also reliability and transparency. Evaluated on a four-class CXR task encompassing COVID-19, pneumonia, TB, and normal findings, CXR-NeXus provides a structured pathway toward clinically auditable and trustworthy deployment.

In this study, our objectives are threefold. First, we aim to improve model interpretability by anchoring predictions to class-specific visual prototypes that represent diverse radiographic phenotypes. Second, we enforce evidence-dependent decision behavior through counterfactual consistency, requiring disease confidence to decrease when salient lesion information is removed. Third, we seek to improve robustness and probability calibration under distributional perturbations, without relying on pixel-level annotations. The primary goal of this work is to promote trustworthy and interpretable behavior under weak supervision, while performance improvements are treated as a secondary but necessary condition for clinical applicability.

## Materials and methods

**Figure 1:**
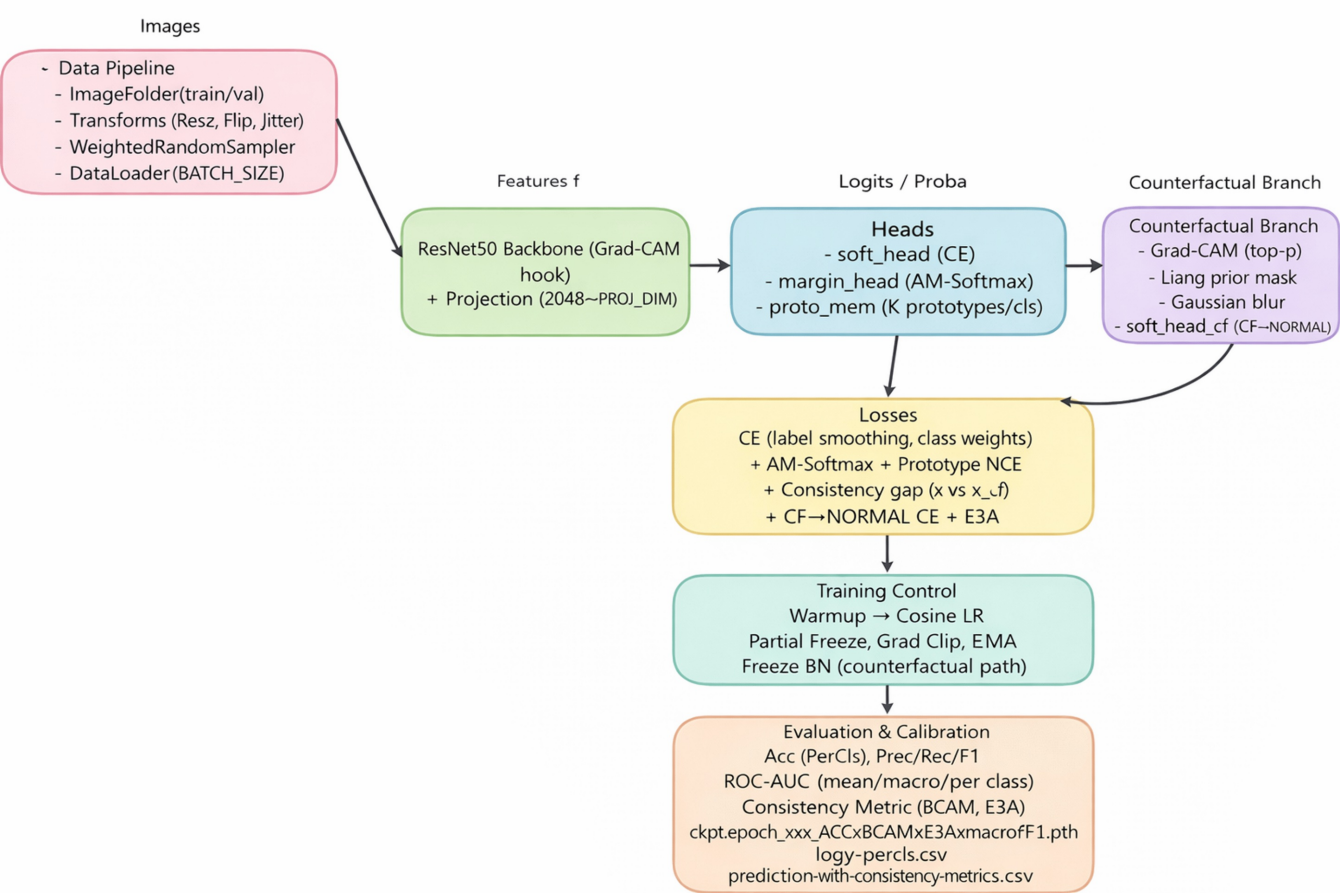
Overview of the proposed CXR-Nexus framework The diagram illustrates the complete training and evaluation pipeline, including data preprocessing, feature extraction, multi-head classification, counterfactual branch design, loss formulation, and evaluation strategy. Image credit: Created by Chiang LF using Graphviz Online [26].

An overview of the proposed CXR-NeXus framework is shown in Figure 1.

The framework is organized as a modular pipeline comprising data preprocessing, feature extraction, classification, counterfactual generation, loss formulation, and training-evaluation control.

In the data preprocessing stage, CXR images from the training and validation sets are loaded using a directory-based ImageFolder structure. During training, images undergo standard augmentations including resizing, random horizontal flipping, and mild brightness perturbation to improve robustness to variations in acquisition conditions and patient positioning. To mitigate class imbalance, a weighted sampling strategy is applied so that each mini-batch contains a balanced representation of the diagnostic categories. Preprocessed samples are then forwarded to the network through a DataLoader interface.

Feature extraction is performed using a convolutional neural network backbone (ResNet-50 (Microsoft Research, Washington, US)). The backbone outputs a high-dimensional feature representation, which is subsequently projected into a lower-dimensional embedding space via a linear projection layer. This embedding serves as a shared representation for downstream classification, prototype matching, and counterfactual analysis. Gradient-based class activation mapping (Grad-CAM) hooks are attached to the final convolutional block to enable the extraction of class-specific saliency maps during training and inference.

The classification module consists of multiple complementary components. A standard softmax head is trained with weighted cross-entropy loss to produce calibrated class probability estimates. In parallel, a margin-based classifier using AM-Softmax is employed to enforce angular separation between classes and improve inter-class discriminability. In addition, a prototype memory module maintains multiple learnable prototypes per class in the embedding space. During training, sample embeddings are encouraged to align with their nearest intra-class prototypes while being repelled from prototypes of other classes, facilitating representation of heterogeneous disease phenotypes.

Counterfactual analysis is implemented as a parallel branch to assess the dependence of predictions on salient image evidence. For each input image, Grad-CAM saliency maps are used to identify the most informative regions. The top-ranked salient regions are combined with a coarse lung-field prior and modified using Gaussian blurring to generate counterfactual images in which lesion evidence is suppressed. These counterfactual images are passed through the same backbone and classification heads as the original inputs.

Model training is guided by a multi-component objective that integrates classification, margin-based separation, prototype consistency, counterfactual constraints, and attention regularization. Optimization is performed using the AdamW optimizer with a cosine annealing learning-rate schedule and an initial warm-up phase. Additional stabilization techniques, including partial layer freezing, gradient clipping, and exponential moving average of model weights, are applied to ensure stable convergence. Model performance is evaluated using standard classification and calibration metrics after training.

## Results

Dataset and experimental setup

All experiments were conducted using the publicly available CXR dataset [25], comprising 7,135 images organized into training, validation, and test subsets. This differentiation was made by following the protocol provided by the original source, with no patient-level overlap between splits. Class distributions were preserved across subsets to mitigate class imbalance effects during evaluation. Each subset included four diagnostic categories: Normal, Pneumonia, COVID-19, and Tuberculosis. Five model configurations were evaluated under identical data splits and training conditions: Baseline-CE, Baseline-CE+AM, Baseline-CE+Proto, Baseline-CE+AM+Proto, and the proposed CXR-NeXus. Models differed only in loss composition and regularization strategies. Performance was assessed using Accuracy, macro-averaged F1 score, macro-Receiver Operating Characteristic - Area Under the Curve (ROC-AUC), Expected Calibration Error (ECE), and Brier score.

Overall classification performance

Across all evaluated configurations, models achieved high classification performance on the four-class CXR task. Summary performance metrics are shown in Figure 1. The baseline cross-entropy model demonstrated strong accuracy and ROC-AUC, indicating a ceiling effect under standard evaluation conditions. Incorporating angular margin constraints and prototype memory preserved high accuracy while improving macro-F1 consistency across classes. The final CXR-NeXus model achieved comparable peak accuracy and ROC-AUC to baseline models while maintaining stable macro-F1 performance, demonstrating that additional regularization did not degrade discriminative capability.

Class-wise error pattern

**Figure 2:**
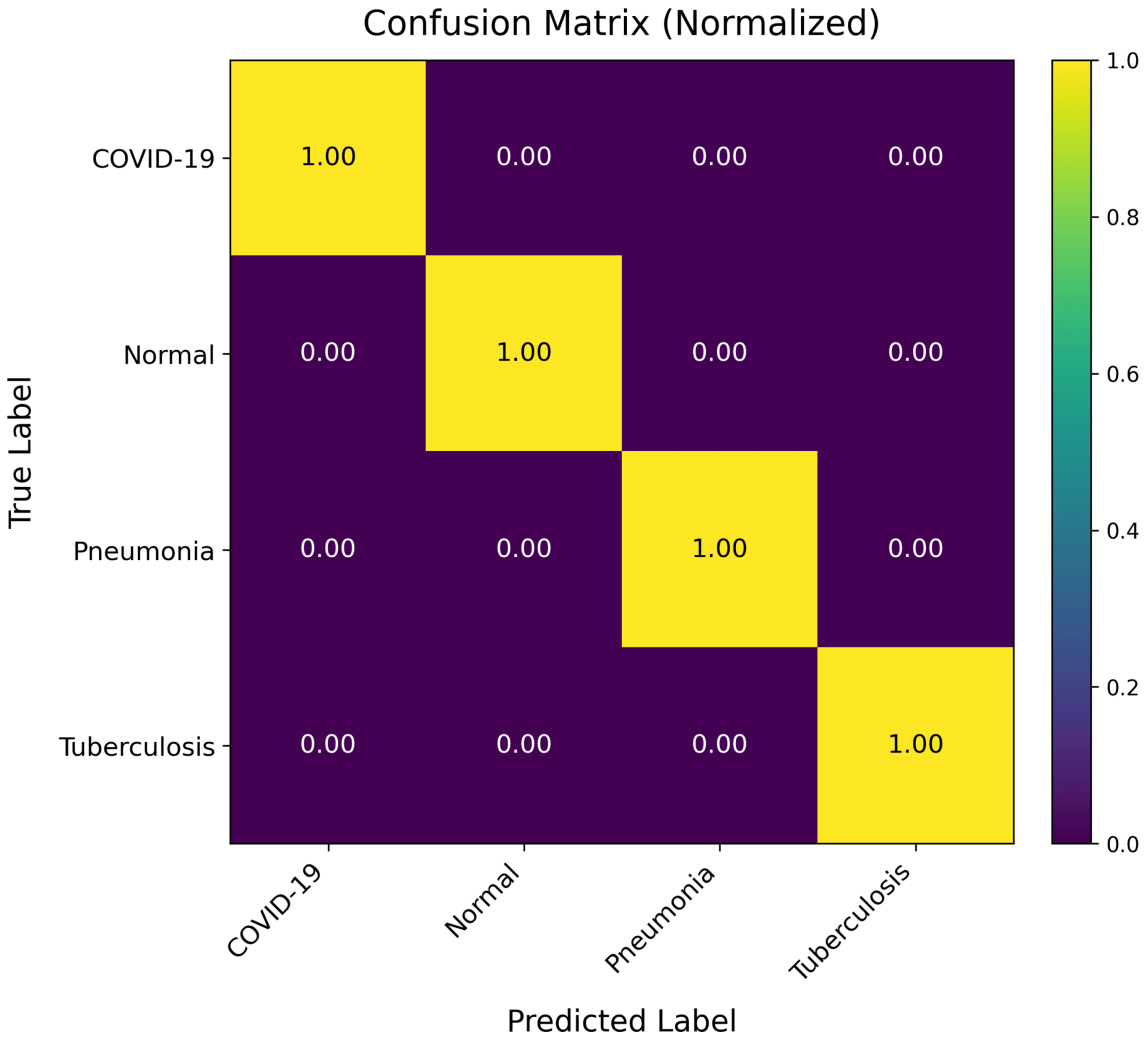
Normalized confusion matrix for four-class chest X-ray classification at the 30th training epoch The matrix summarizes the classification performance of CXR-NeXus across COVID-19, Pneumonia, Tuberculosis, and Normal categories. Diagonal elements represent correct predictions, while off-diagonal entries indicate misclassifications. The results demonstrate strong separability among pathological classes, with the majority of errors occurring between Normal and Pneumonia, reflecting inherent visual overlap in mild or early-stage findings.

Figure 2 presents the normalized confusion matrix of the final CXR-NeXus model evaluated on the independent test set.

Strong diagonal dominance was observed across all four categories, indicating effective class-wise discrimination. COVID-19 cases were classified with high accuracy, with minimal confusion with other infectious diseases. Pneumonia and Tuberculosis classes also demonstrated low cross-category confusion. The majority of misclassifications occurred between Normal and Pneumonia, reflecting overlap between mild inflammatory findings and near-normal radiographic appearances. No systematic bias toward a dominant class was observed.

Robustness under progressive perturbation severity

**Figure 3:**
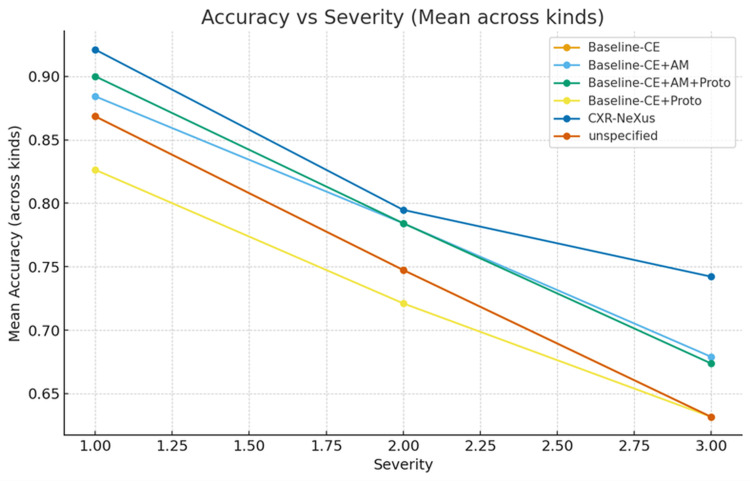
Variation of average classification accuracy under different perturbation levels The figure shows the average classification accuracy across all diagnostic categories as perturbation severity increases from mild (S1) to severe (S3). All models exhibit performance degradation with increasing perturbation strength; however, CXR-NeXus consistently maintains higher accuracy across all severity levels compared with baseline configurations. Severity levels represent progressively stronger combinations of image distortions, including brightness variation, contrast shifts, blur, noise, and cropping.

Model robustness was evaluated under increasing perturbation severity levels (S1-S3), as shown in Figure 3.

All models exhibited declining accuracy with increasing perturbation strength. However, the magnitude of degradation varied among configurations. CXR-NeXus consistently maintained higher average accuracy across all severity levels, achieving approximately 0.91 at S1, 0.79 at S2, and 0.74 at S3. In contrast, baseline models showed steeper declines, with accuracy values near or below 0.67 at S3.

**Figure 4:**
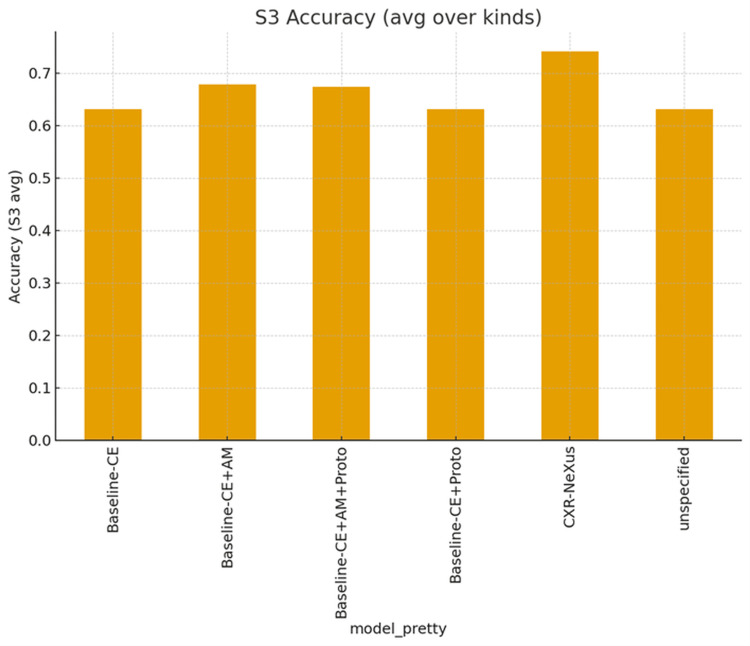
Average classification accuracy under the most severe perturbation condition (S3) The figure compares mean accuracy across all perturbation types for different model variants. CXR-NeXus achieves the highest accuracy under S3 conditions, indicating superior robustness to severe image degradation compared with baseline and ablation models.

Figure 4 summarizes performance under the most severe perturbation condition (S3).

CXR-NeXus achieved the highest aggregated accuracy among all configurations. Models incorporating angular margin constraints outperformed those trained with cross-entropy alone, while prototype-only extensions demonstrated limited robustness under severe distortion.

Calibration performance under severe perturbations

**Figure 5:**
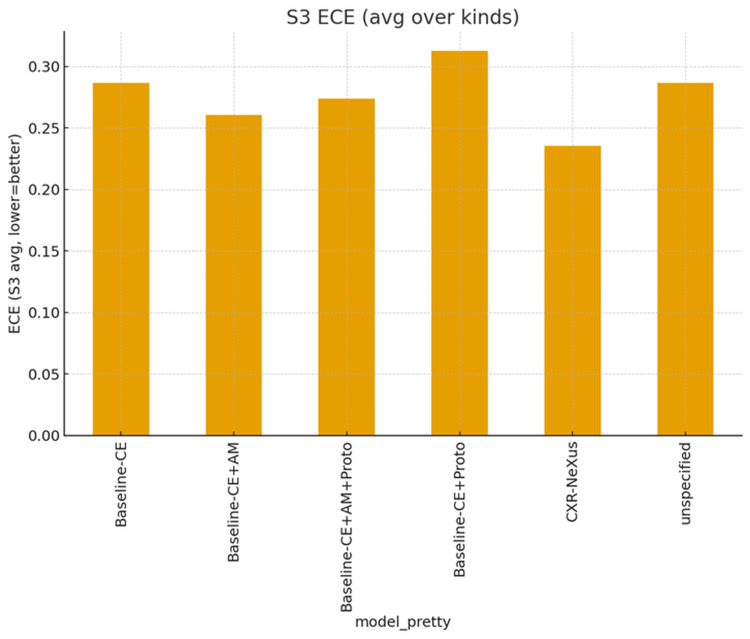
Probability calibration error (ECE) under the most severe perturbation condition (S3) Lower Expected Calibration Error (ECE) values indicate better probability calibration. CXR-NeXus demonstrates the lowest calibration error among all the compared models under severe perturbations, suggesting more reliable confidence estimation when visual evidence is substantially degraded.

Calibration quality under S3 conditions is shown in Figure 5.

CXR-NeXus exhibited the lowest ECE, indicating improved alignment between predicted probabilities and observed outcomes. Baseline models demonstrated higher ECE values, particularly those lacking margin-based or counterfactual regularization. Similar trends were observed for Brier score, with CXR-NeXus consistently yielding lower error values under severe perturbation.

Perturbation-type-specific analysis

Perturbation severity levels (S1-S3) correspond to progressively stronger combinations of image distortions, including increasing ranges of brightness and contrast variation, larger Gaussian blur kernels, higher noise variance, and more aggressive cropping and zooming.

**Figure 6:**
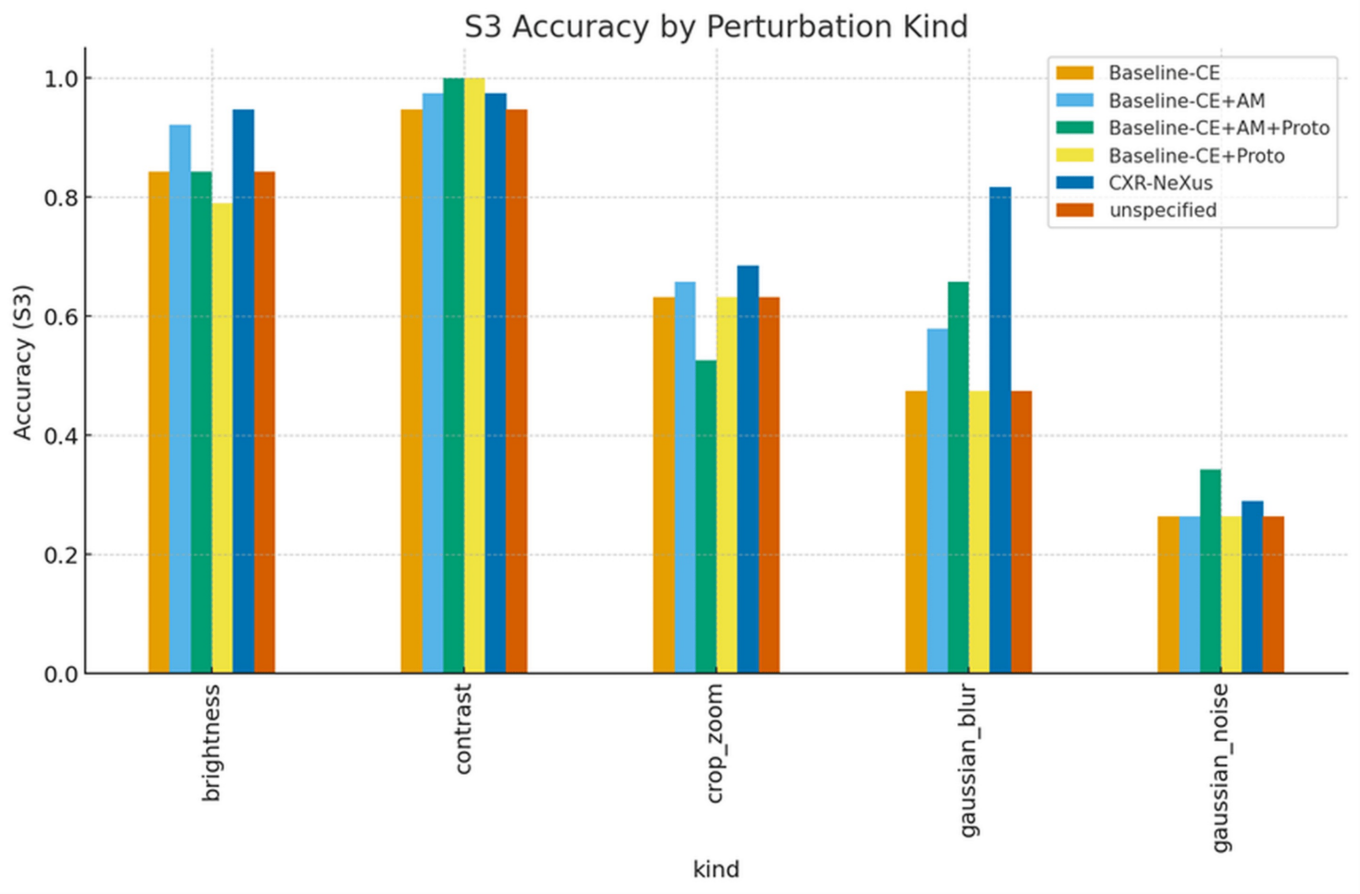
Average accuracy under S3 conditions, grouped by perturbation type Classification accuracy under the most severe perturbation condition (Severity 3, S3) stratified by perturbation type. Accuracy is reported for brightness, contrast, cropping/zooming, Gaussian blur, and Gaussian noise across all model configurations. CXR-NeXus consistently demonstrates higher accuracy than baseline models, particularly under spatially destructive perturbations such as Gaussian blur and cropping/zooming, indicating enhanced robustness to severe image degradation.

**Figure 7:**
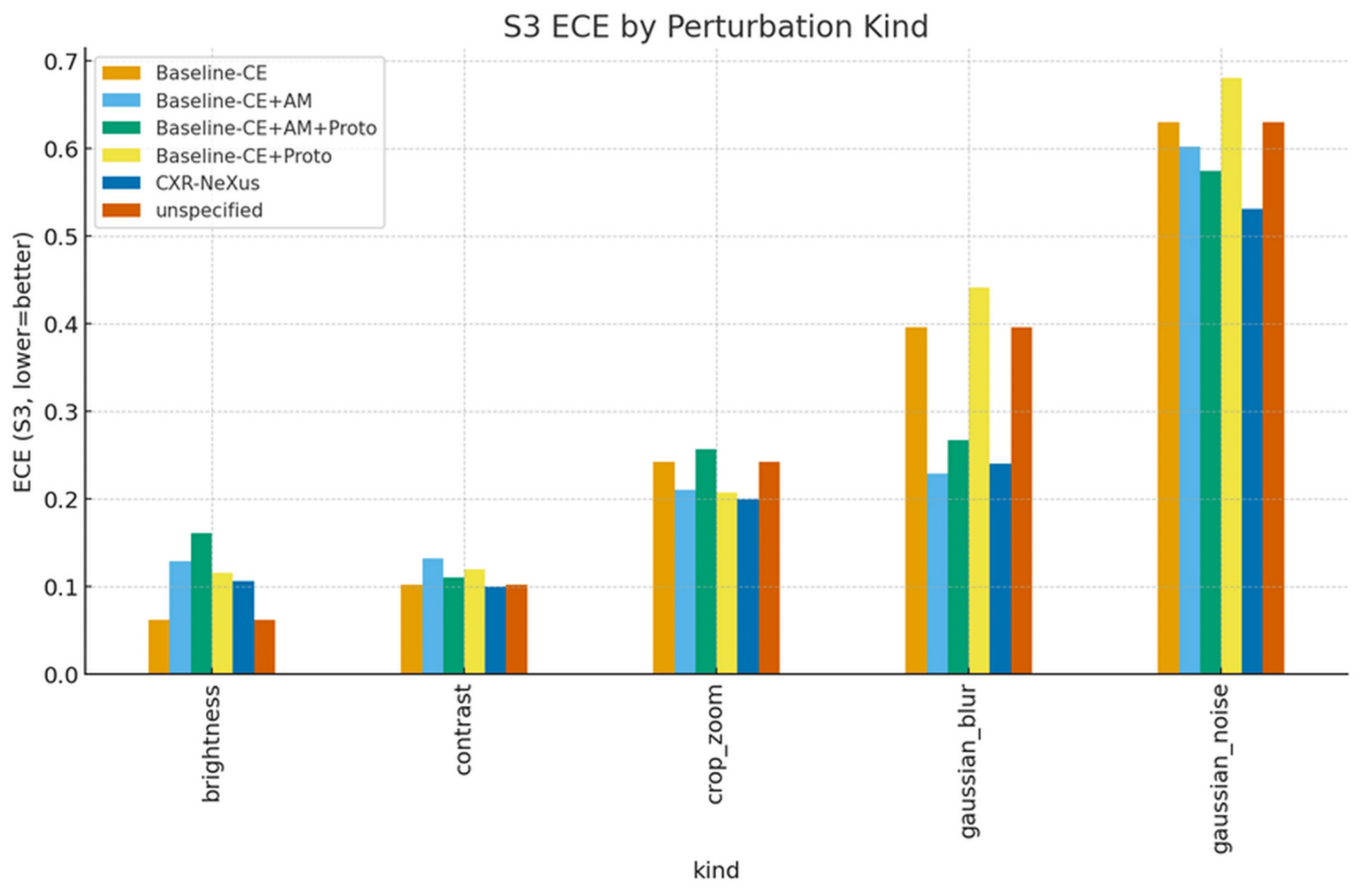
Probability calibration error grouped by perturbation type under S3 conditions Expected Calibration Error (ECE) under the most severe perturbation condition (Severity 3, S3) grouped by perturbation type. Lower ECE values indicate better probability calibration. Across brightness, contrast, cropping/zooming, Gaussian blur, and Gaussian noise perturbations, CXR-NeXus consistently exhibits lower calibration error compared with baseline models, reflecting improved reliability of predicted confidence under severe image degradation.

**Figure 8:**
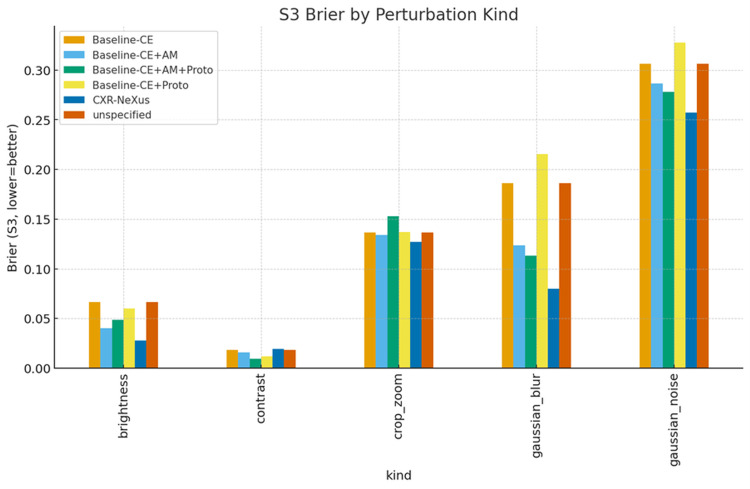
Brier score grouped by perturbation type under S3 conditions Brier score under the most severe perturbation condition (Severity 3, S3) across different perturbation types. Lower Brier scores indicate better probabilistic accuracy. CXR-NeXus consistently achieves lower Brier scores than baseline models across brightness, contrast, cropping/zooming, Gaussian blur, and Gaussian noise, demonstrating superior probability calibration and reduced overconfidence under severe image degradation.

Figures 6-8 present accuracy, ECE, and Brier score grouped by perturbation type under S3 conditions.

Under brightness and contrast perturbations, all models maintained relatively high accuracy, with CXR-NeXus achieving the most favorable combination of accuracy and calibration. Under Gaussian blur, CXR-NeXus retained substantially higher accuracy compared with baseline configurations. Gaussian noise resulted in marked performance degradation across all models, although CXR-NeXus demonstrated modestly improved calibration relative to cross-entropy baselines. Under cropping and zooming perturbations, CXR-NeXus again showed superior stability in both accuracy and probability calibration.

Test set misclassification analysis

Detailed misclassification statistics on the test subset are summarized in Table 1.

**Table 1:** Misclassification analysis on the test subset Test subset from [25].

True label	Total samples	Misclassified as	Misclassification count	Misclassification rate (%)
COVID-19	106	Tuberculosis	1	0.94
Normal	234	Pneumonia	51	21.79
Pneumonia	390	Normal	7	1.79

Among COVID-19 cases, only one out of 106 images was misclassified, yielding an accuracy exceeding 99%. Pneumonia cases showed low misclassification rates, with only seven of 390 images predicted as Normal. The Normal category exhibited the highest misclassification rate, with 51 of 234 images classified as Pneumonia. This pattern represents the dominant source of error and reflects diagnostic ambiguity in radiographs with subtle or borderline findings. Overall, misclassification between infectious disease categories remained limited, supporting stable class separation across clinically relevant conditions.

## Discussion

In this study, we proposed and systematically evaluated CXR-NeXus, a counterfactual- and evidence-aligned deep learning framework designed to improve not only classification accuracy but also decision stability, calibration, and interpretability in multi-class CXR diagnosis. Prior CXR deep learning systems have primarily focused on maximizing predictive performance on curated datasets, often without explicit constraints on how decisions are formed or how confidence behaves under distributional shifts [1-5,9,10]. In contrast, CXR-NeXus explicitly constrains decision formation through counterfactual consistency, evidence-aligned attention, and angular-margin learning, addressing growing concerns regarding robustness, calibration, and trustworthiness in medical AI systems [16,20,21]. The experimental results demonstrate that these design choices yield consistent advantages over baseline models across heterogeneous perturbation environments, particularly in terms of robustness and probabilistic reliability.

Importantly, the primary contribution of this work lies not in introducing a novel architectural building block but in unifying multiple interpretability and robustness mechanisms into a single training objective. Unlike prior approaches that apply prototypes, saliency maps, or counterfactual analysis as post-hoc interpretability tools [17,18,27], CXR-NeXus integrates angular-margin learning, prototype anchoring, counterfactual confidence modulation, and evidence-aligned attention directly into the optimization process. This joint formulation constrains not only what the model predicts, but how decisions and confidence are formed, yielding more predictable and auditable behavior under evidence perturbation [28].

A key observation from the results is that under perturbation-free or mildly perturbed conditions, most model variants -including simple cross-entropy baselines - approach performance saturation. This “ceiling effect,” commonly reported in curated CXR benchmarks such as ChestX-ray8, CheXpert, and PadChest, limits the discriminative value of standard metrics such as accuracy or ROC-AUC when models are evaluated only under ideal conditions [9-12]. Similar saturation phenomena have been observed in prior comparative studies of chest radiograph classifiers, where architectural differences yield marginal gains under clean conditions but diverge under stress testing [5,16]. However, when perturbation severity increases, clear and clinically meaningful differences emerge. Across severity levels S1 to S3, CXR-NeXus consistently exhibits a slower degradation in accuracy and a smaller increase in calibration error compared with all baseline configurations, suggesting improved stability under distribution shift.

The robustness gains observed in CXR-NeXus can be attributed to the joint action of its architectural and training constraints. Angular-margin learning, such as AM-Softmax, has been shown to improve inter-class separability and decision boundaries in both natural and medical image classification tasks [14,18]. This partially explains the improved performance of AM-based baselines under moderate perturbations. Prototype memory further enhances representational consistency by anchoring features to class-specific visual prototypes, a strategy previously demonstrated to improve interpretability and intra-class heterogeneity modeling [18,19]. However, as observed in our experiments, prototype-only variants exhibit vulnerability under cropping and geometric distortions, consistent with prior findings that prototype mechanisms may overfit fixed morphological templates [19]. CXR-NeXus mitigates this limitation by integrating counterfactual consistency and evidence-aligned attention, forcing the model to reassess its predictions when salient lesion evidence is suppressed.

Counterfactual consistency plays a central role in shaping causal decision behavior. Prior work has shown that counterfactual reasoning can reveal and reduce reliance on spurious correlations in deep models, particularly in medical imaging contexts where shortcuts are prevalent [20-23]. By requiring that disease confidence decreases when lesion evidence is removed, CXR-NeXus discourages exploitation of confounders such as text markers, borders, or scanner-specific artifacts, which have been documented as failure modes in CXR models [16,24]. Importantly, this constraint is implemented as a logit-gap regularization rather than hard relabeling of counterfactual samples, aligning with recent calibration-based counterfactual training strategies that avoid inducing class imbalance bias or decision collapse [21]. The improved stability observed under severe blur and cropping perturbations supports the effectiveness of enforcing evidence-dependent confidence modulation.

Mechanistically, counterfactual consistency regularization encourages a controlled reduction in disease confidence when salient lesion evidence is suppressed, preventing overconfident predictions driven by spurious cues. When combined with angular-margin learning, this constraint sharpens class boundaries while stabilizing confidence estimates near decision margins. This interaction provides a plausible explanation for the consistently lower ECE and Brier scores observed under severe perturbations.

Evidence-aligned attention further enhances interpretability and robustness by constraining saliency distributions to anatomically plausible lung regions. Gradient-based saliency methods such as Grad-CAM have been widely used to interpret CXR models, but prior studies have highlighted their susceptibility to attention leakage and post-hoc inconsistency [17,20]. By incorporating attention alignment as a training constraint rather than a visualization tool, CXR-NeXus follows emerging paradigms that treat interpretability as a form of regularization [20]. The consistent reduction in calibration error and Brier score observed for CXR-NeXus under perturbations indicates that attention regularization improves not only spatial plausibility but also probabilistic trustworthiness. Well-calibrated probabilities are critical for clinical deployment, as miscalibrated confidence can undermine threshold-based decision-making and risk stratification [21,24].

Analysis of perturbation-specific behavior provides additional insights into model characteristics. Under global intensity perturbations such as brightness and contrast shifts, most models maintain relatively high accuracy, reflecting the known resilience of convolutional backbones to uniform gain changes [5,16]. In contrast, spatially disruptive perturbations - including Gaussian blur, noise, and cropping - expose substantial performance divergence. CXR-NeXus demonstrates superior resistance to blur and cropping, indicating a greater reliance on region-level structural evidence rather than high-frequency texture cues, a distinction increasingly recognized as critical for medical image robustness [16,24]. Under extreme Gaussian noise, performance degrades for all models, consistent with prior observations of shared vulnerability to high-frequency stochastic interference in chest radiograph classifiers [5,16]. Although prototype-enhanced models show partial advantage in this regime, calibration errors remain elevated, suggesting opportunities for future integration of noise-aware or frequency-domain regularization.

Confusion matrix analysis provides additional clinical context. The model demonstrates near-perfect discrimination for COVID-19 cases, reflecting the relatively distinctive radiographic patterns present in curated COVID-19 CXR datasets [7,14,16]. The dominant source of error arises from Normal cases misclassified as Pneumonia, a pattern consistent with known diagnostic ambiguity in chest radiography. Prior clinical and dataset analyses have reported substantial overlap between mild inflammatory findings and early pneumonia, even among expert radiologists [3,7,16]. Rather than indicating a structural deficiency, this behavior underscores the inherent uncertainty of borderline cases in weakly supervised CXR datasets. Such confusion patterns have been widely reported even among expert readers and are a known characteristic of report-derived supervision, suggesting that these errors reflect diagnostic ambiguity rather than a failure of evidence localization. Importantly, the model exhibits low confusion between major pathological classes, supporting the conclusion that CXR-NeXus learns meaningful disease-specific representations rather than superficial correlations. 

From a broader perspective, the primary contribution of this work lies not in introducing a novel neural building block but in integrating interpretability, causality, and stability directly into the training objective. Prior efforts in CXR analysis have largely treated interpretability as a post-hoc analysis step [17], whereas more recent work has argued for embedding explanatory constraints directly into learning [20,21]. CXR-NeXus operationalizes this principle by jointly aligning counterfactual behavior, prototype structure, angular margins, and anatomical priors. This reframing of interpretability as regularization moves medical AI beyond opaque accuracy optimization toward models that justify their decisions, maintain calibrated confidence, and behave predictably under uncertainty.

Several limitations of this study should be acknowledged. First, the evaluation was conducted on a single CXR dataset, and although extensive perturbation testing approximated domain shifts, external multi-institutional validation remained necessary to confirm generalizability, as emphasized in prior dataset bias analyses [11,16]. Second, perturbations are synthetically generated and may not fully capture real-world acquisition variability such as hardware differences or patient positioning artifacts [16]. Third, while evidence-aligned attention improves plausibility, it relies on coarse lung priors rather than precise anatomical segmentation, a limitation shared by many large-scale CXR studies [9-12]. In addition, interpretability in this study is assessed indirectly through model behavior, attention plausibility, and calibration trends, rather than through formal human-rated explanation studies or quantitative localization benchmarks. Future work may integrate learnable or multimodal anatomical constraints and formal explanation evaluation protocols to further refine spatial reasoning and assess explanation fidelity more rigorously. Finally, the current framework focuses on image-level classification; extending counterfactual and evidence-aligned principles to localization or longitudinal prediction tasks represents an important direction for future research [24].

In summary, CXR-NeXus demonstrates that enforcing evidence-dependent decision rules - rather than solely optimizing predictive scores - leads to models that are more robust, better calibrated, and more interpretable. By explicitly constraining how confidence should change when evidence is altered, the framework establishes a causal link between visual features and diagnostic decisions, aligning with emerging calls for trustworthy and clinically reliable medical imaging AI [16,20,21,24]. These properties are essential for real-world deployment, where transparency, stability, and calibrated uncertainty are as critical as accuracy.

## Conclusions

In this study, we introduced CXR-NeXus, a multi-class CXR classification framework designed to enhance not only predictive performance but also robustness, calibration, and interpretability. By integrating angular-margin learning, prototype memory, counterfactual consistency, and evidence-aligned attention into a unified training objective, the proposed model explicitly constrains how diagnostic decisions are formed and how confidence responds to changes in pathological evidence.

Experimental evaluations under heterogeneous perturbation conditions demonstrate that CXR-NeXus maintains more stable accuracy and improved probability calibration compared with baseline models, particularly in the presence of severe image degradation. Beyond performance metrics, the framework provides interpretable decision traces through anatomically plausible attention maps and prototype-based feature anchoring, supporting transparent and auditable inference.

These findings suggest that enforcing evidence-dependent behavior during training can meaningfully improve the reliability of medical imaging models. CXR-NeXus represents a step toward clinically trustworthy artificial intelligence systems that not only achieve high accuracy but also sustain stable, interpretable, and well-calibrated decisions under uncertainty.
